# Effectiveness of introducing point of care capillary testing and linking screening with routine appointments for increasing blood lead screening rates of young children: a before-after study

**DOI:** 10.1186/s13690-015-0111-y

**Published:** 2015-12-29

**Authors:** Frances Boreland, David Lyle, Anthony Brown, David Perkins

**Affiliations:** Broken Hill University Department of Rural Health, University of Sydney, Broken Hill Centre for Remote Health Research, Corrindah Court, PO Box 457, Broken Hill, 2880 NSW Australia; School of Rural Health. University Of Sydney, 11 Moran Drive, Dubbo, 2830 NSW Australia; Centre for Rural and Remote Mental Health, University of Newcastle, c/o Bloomfield Hospital Forest Road, Orange, 2800 NSW Australia

**Keywords:** Children, Screening, Blood lead levels, Attendance, Monitoring

## Abstract

**Background:**

Lead has significant neuro-toxic effects, particularly for young children. Voluntary screening of pre-school aged children for elevated blood lead levels has been an important part of the lead management program in the mining town of Broken Hill (NSW, Australia) since 1991, where lead remains a significant public health issue for young children despite average blood lead levels having fallen by two-thirds. The annual proportion of children screened declined to 0.39 in 2008. The objective of this study was to determine the impact of changing to capillary screening and linking screening with existing routine health programs on participation in blood lead screening by young children in the community.

**Methods:**

We used a before-after study. Screening rates were determined from routinely collected service data and analysed using cross-sectional and cohort analyses.

**Results:**

The proportion of children screened annually increased from 0.39 in 2008 to 0.75 in 2012, with the greatest increases among 11–23 and 48–59 month old children. The proportion of children screened at least once by 24 months increased from 0.63 for children born in 2007 to 0.98 for children born in 2010. Attendance stabilized after capillary screening was introduced, and increased markedly after screening was offered at immunization.

**Conclusons:**

Changing from venous to capillary screening stabilized attendance and improving convenience was associated with dramatically increased screening. Linking screening with well-accepted mainstream child health programs is an effective strategy to improve participation in blood lead screening programs. The findings have implications for improving participation in other health screening programs.

## Background

Lead has significant neuro-toxic effects, particularly for young children [1, 2]. Emerging evidence about the effects of chronic low blood lead levels highlights the importance of a systemic approach to reducing community lead exposure [3–8]. In response there is increasing emphasis on primary prevention and several countries have reduced their blood lead thresholds for investigation of an individual’s source of exposure from 10 to 5 μg/dL or lower [3, 9–12].

Screening at-risk populations is an important component of lead control programs, both detecting individuals who may benefit from available advice and interventions, and monitoring the effectiveness of community-wide efforts to reduce blood lead levels [9, 13, 14]. To succeed screening programs must be both acceptable and accessible to the community. As with other health issues for which screening is appropriate, blood lead screening is more acceptable when it is less invasive, easily accessible, and when people believe something can be done about the health issue if a problem is detected [15]. Participation in blood lead screening programs varies widely, and may be very difficult to increase [15–18].

Voluntary screening of pre-school aged children for elevated blood lead levels has been an important part of the lead management program in Broken Hill (NSW, Australia) since 1991, when lead was identified as an important community public health issue [19]. The average blood lead level of 1–4 year old children has fallen by 67 % since 1991. However, lead is still an important public health issue for the local community, with 21 % of children screened in 2012 having blood lead levels of 10 μg/dLor higher [1, 20].

During the 1990s the proportion of eligible children screened each year varied markedly: 0.50–0.65 were screened most years; screening peaked at 0.72 in 1994 and 0.73 in 1998 in response to more intensive program activity and reached a low of 0.39 in 1993 when program activity was minimal due to restricted funding [19]. The lead management program initially operated as a separate entity, but in 2001 it was integrated with mainstream services to ensure its sustainability; subsequently funding decreased significantly. Attendance at screening declined steadily, from 0.56 in 2001 to 0.39 for all 1–4 year old children in 2008; and to 0.25 for Indigenous children in 2009 [19–21]. Community consultations identified several possible barriers to attending screening including parental perceptions that the collection of the blood sample caused discomfort to children and access to the weekly screening clinic was inconvenient, and highlighted the importance of screening occurring within a culturally acceptable model [22, 23].

The aim of this study is to evaluate the effectiveness of changes to the blood lead screening protocol to examine:

1. the impact of the changes on cumulative and overall blood lead screening rates and service provision, and the relative importance of changing the screening method and improving access;
2. whether all children have benefited from the changes and if further outreach is required for any particular group;
3. the effect of changing to capillary screening on the estimate of the underlying mean blood lead level.

## Method

### Study design

A before-after study based on routinely collected data.

### The intervention

Capillary screening using the LeadCare II was introduced in October 2008 and subsequently linked with existing well-accepted programs to improve accessibility and convenience for parents as described below. From 2010 screening was incorporated into routine healthy children checks at the local Indigenous health service (rather than being available once or twice a year). From 2011 screening was offered when children attended the mainstream community health service for immunization (offered at 18 month immunization from March and for all immunizations—12 months, 18 months and 4 years—from September). Text message (SMS) appointment reminders were also introduced by the mainstream health service in May 2011. Confirmatory venous tests are offered for blood leads ≥15 μg/dL.

The LeadCare II is a point of care testing device for analyzing whole blood lead samples; it received a Clinical Laboratory Improvement Ammendment of 1988 (CLIA) waiver in 2006. It was the only commercially available point of care testing euipment for measuring blood lead levels. FDA has approved this technology for blood lead testing in non-traditional laboratory settings as long as appropriate training and finger cleaning procedures are instituted [24].

### Data sources

Numerator data for all analyses were obtained from routinely collected data extracted from the Lead Management Program ACCESS database, which contains information on all blood lead tests conducted at both the mainstream and Indigenous health clinics. Data were extracted for all 7–59 month old children who had a blood lead test between 1/1/2005 and 31/12/2012 (approximately 10,100 records) and were resident in Broken Hill at the time. The items extracted were: birth date, sex, date of test, address, area of town and numeric identifier (assigned at first blood lead test). Address was used to code for one of five lead risk zones within Broken Hill and then removed from the dataset to protect confidentiality. Thirteen records (six children) were excluded from further analysis because the child’s residential address could not be confirmed (for example, was listed as a post office box) and 11 records (five children) because sex was not documented. This left data on 2287 children for analysis.

The number of children usually resident in each locality was used as the denominator for calculating annual attendance rates and was estimated using 2006 and 2011 Australian Bureau of Statistics (ABS) census data [25, 26]. Change between the two censuses was assumed to be linear. The number of children born to mothers resident in Broken Hill was used as the denominator for cohort attendance rates and was estimated from the NSW Midwives Database (NMD) (Centre for Epidemiology and Research, NSW Ministry of Health). Analysis was conducted using Microsoft Excel 2010 and SAS 9.4.

### Analysis

To assess the impact of changes to the screening protocol on cumulative attendance rates, we calculated the proportion of children screened at least once by 12, 18, 24, 48 and 59 months for each annual birth cohort between 2005 and 2011.

To examine the effect of the changes on service provision, all available screening data was used to calculate the number of days screening was available each calendar year and the average number of children screened each day.

We examined the change in overall attendance after the new screening method was introduced and after screening was linked with immunization and SMS reminders introduced. To facilitate comparison with previous publications [19, 27] this analysis was restricted to 12–59 month old children. If a child was screened more than once in a calendar year, only the first test was included to avoid double counting. Piecewise linear regression was used to allow (and test for) change in slope at chosen cut-points to compare the trend in the proportion of children screened during three periods: 2005–2008 when venous screening was available; 2008–2010 when capillary screening was available and 2010–2012 when screening was linked with immunization and text reminders introduced. The significance of the change in proportion of children screened at the beginning and end of each period was assessed by calculating the difference in proportions and associated 95 % confidence intervals.

To assess whether all children in the community have benefited from the changes to the screening protocol and if further outreach is required for any particular group, we estimated the proportion of 12–59 month old children screened in a given year and used stratified analysis to examine the impact of age, gender, locality and year of testing on attendance rates. Difference in proportions and associated confidence intervals were used to compare attendance at particular time points. For these analyses, tests occurring before a child’s first birthday were excluded, and if a 12–59 month old child had more than one test in a calendar year only the first test was used. The two areas with the highest environmental lead risk (zones 1 and 2) had small numbers of children, and were combined for all analyses to improve the robustness of estimates.

Because capillary screening returns slighlty higher values than venous, we calculated geometric mean and 95 % confidence intervals for 12–59 month old children for 2005–2012 to determine whether there was a consistent difference in mean after the change to capillary testing. Geometric means were used because the blood lead values were not normally distributed without the logarithimic transformation. As a further check we also calculated the annual proportion of children with test results equal to or greater than 5 and 10 μg/dL.

#### Ethical approval

The study was approved by the Greater Western Area Health Service Human Research Ethics Committee (Reference No. HREC/11/GWAHS/7) and the approval was accepted by the Executive Committee of the University of Sydney Human Research Ethics Committee (Reference No. 13716).

## Results

### Attendance by birth cohort

**Fig. 1:**
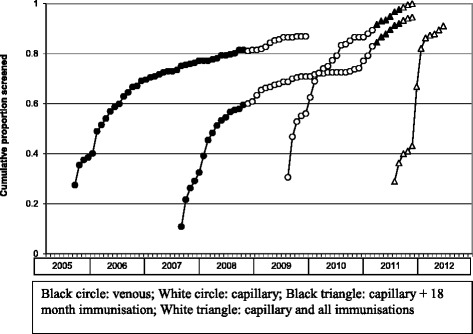
Estimated cumulative proportion of Broken Hill children having their blood lead levels screened at least once for specific annual birth cohorts (2005, 2007, 2009 and 2011)

The change to capillary screening was associated with a marked increase in screening among children who were less than 12 months old when the change was introduced (Fig. 1). The proportion of children screened by 12 months was 0.39 for children born in 2007 compared with 0.60 for children born in 2008 (0.21 increase; 95 % confidence intervals increase of 0.12–0.29). However, screening rates of children who were older when capillary screening was introduced did not increase. For example, children born in 2007, who were 10–22 months when capillary screening was introduced in October 2008, had slightly lower cumulative screening rates by 24 months than children born in 2006 (0.63 and 0.69 respectively, 0.06 reduction in screening, 95 % confidence intervals increase of 0.14 reduction to 0.03 increase). Estimated cumulative attendance for screening was slightly above 100 % for children born in 2008, 2009 and 2010 (106, 105 and 105 % respectively).

Offering screening when children attended for immunization markedly increased screening rates. The proportion of children screened at least once by 24 months increased from 0.63 for children born in 2007 to 0.98 for children born in 2010 (0.34 increase in screening, 95 % confidence intervals increase of 0.28–0.41).

### Service availability

The number of days when screening was available more than doubled after screening was offered to children attending for immunization, from 80 screening days in 2010 to 184 screening days in 2012 (Table 1). Although the average number of children screened per screening day decreased by a quarter during the same period, the number of children screened per year increased by 70 %.

**Table 1:** Estimated birth cohorts, attendance at blood lead screening by 12–59 month old children in Broken Hill, 2005–2012: number screened, number of days on which screening was provided, and average number of children screened per day

	2005	2006	2007	2008^a^	2009	2010^b^	2011^c^	2012
Estimated birth cohort (NMD)	237	218	240	236	216	228	190	-
Children screened	563	456	456	361	376	397	554	665
Number of screening days	53	46	53	72	76	80	148	184
Average number children screened per screening day	10.34	9.76	8.66	5.01	4.75	5	4	3.66

^a^Capillary introduced October 2008

^b^Screening regularly available at local indigenous health clinic

^c^Screening offered at 18 month immunization in March and at all immunizations from September; SMS appointment reminders introduced in May

### Overall screening participation

**Fig. 2:**
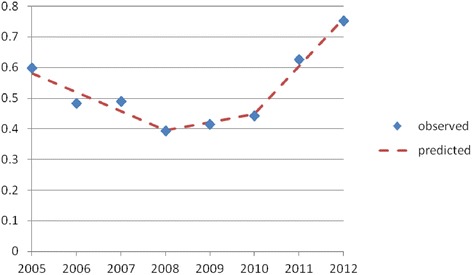
Estimated proportion* of 12–59 month old children screened for lead in Broken Hill before and after the introduction of screening by capilliary in October 2008 and offering screening with immunisation from 2011

Overall participation of 12–59 month old children in screening showed three distinct phases (Fig. 2). From 2005 to 2008 the proportion of children screened annually decreased from 0.59 in 2005 to 0.39 in 2008 (decrease = 0.29; 95 % CI decrease of 0.16–0.25) and regression analysis estimated attendance was falling steadily at about 6 % per year: y = 0.58207–0.06197, P =0.0068; where the intercept (0.58207) gives the predicted proportion screened in 2005 and the coefficient for year (−0.06197) gives the expected change per year from 2005 to 2008. After capillary screening was introduced the proportion of children screened increased slightly from 0.39 in 2008 to 0.44 in 2010 (increase of 0.05; 95 % CI increase of 0.00–0.09) and regression analysis confirmed the underlying trend had changed direction, with attendance predicted to increase by about 9 % per year from 2008 to 2010: y = 0.58207–0.06197 + 0.08782; P =0.0288. Attendance increased markedly when screening was linked with immunization and SMS reminders were introduced, rising to 0.75 in 2012 (increase of 0.31, 95 % CI increase of 0.27–0.35) and regression analysis confirmed a significant change in underlying trend, with attendance predicted to increase by 13 % per year for 2010–2012: y = 0.58207–0.06197 + 0.08782 + 0.13048; P =0.0158.

### Locality, age and gender specific attendance rates

At the beginning of the study period children living in the highest lead risk zones were more likely to attend than children living in other areas (0.90 vs 0.51 %–0.57; difference = 0.33, 95 % CI difference of 0.26–0.40) but their attendance declined more rapidly and by 2010 was similar to that of children living in other areas (0. 48 vs 0.33–0.46). Attendance increased for children living in all areas of town after screening was linked with immunization, but increased more slowly for children living in the highest lead risk districts; so that by 2012 their attendance was midrange (0.71 vs 0.64–0.80).

One-year-old (i.e., 12–23 months) children consistently had the highest annual attendance rates across the study period. Prior to the introduction of capillary screening this ranged from 0.75 in 2005 to 0.53 on 2008. Attendance increased markedly after screening was offered with the 18-month immunization in 2011. By 2012, 1.00 (238, from an estimated population of 233) of 1-year-olds were screened. Four-year old children consistently had lower screening rates than younger children (0.24–0.43 vs 0.37–0.59 for 3-year olds and 0.43–0.66 for 2-year-olds) until screening was offered with the 4-year-old immunization in 2011, after which their attendance increased markedly and slightly exceeded that of 2 and 3 year olds (0.73 vs 0.61–0.62).

In most years screening rates were 0.08–0.10 higher for boys than girls; both showed similar changes in attendance over time.

### Effect of changing to capillary method on estimate of population blood lead levels

There was no evidence that changing to capillary sampling affected the estimate of the underlying population blood lead level (Table 2). The mean blood lead level did not increase after capillary screening was introduced, but continued to fluctuate at or slightly below levels observed during the first 3 years of the study period (when only venous sampling occurred). Similarly there was no consistent change in the proportion of children screened who had blood lead levels equal to or above 5 or 10 μg/dL (Table 2).

**Table 2:** Annual blood lead screening metrics, Broken Hill 2005–2012; 12–59 month old children

	2005	2006	2007	2008	2009	2010	2011	2012
Children screened	563	456	456	361	376	397	553	665
	0.60	0.48	0.49	0.39	0.41	0.44	0.62	0.75
Geometric mean (μg/dL)	5.7	6.1	5.9	4.8	5.8	4.7	4.8	5.4
Lower 95 % CI (μg/dL)	5.4	5.7	5.5	4.5	5.5	4.4	4.6	5.2
Upper 95 % CI (μg/dL)	6.1	6.5	6.3	5.2	6.2	5.0	5.0	5.7
Proportion children with blood leads equal to or greater than 5 μg/dL	0.64	0.69	0.66	0.52	0.61	0.40	0.45	0.52
Proportion children with blood leads equal to or greater than 10 μg/dL	0.37	0.40	0.40	0.32	0.34	0.33	0.27	0.39

## Discussion

The changes to the screening protocol were associated with a significant increase in overall annual screening participation, from 0.39 in 2008 to 0.75 in 2012. This exceeded the program’s previous highest participation rates (72 and 73 %), achieved in 1994 through a community-wide doorknock and in 1998 through heightened program activity [19]. Importantly, attendance has increased for children in all age groups, living in all areas of town and for both boys and girls. The increased attendance has been maintained, reaching 0.78 in 2013 [27]. By 2012 the program met local targets for screening and recommendations for universal screening in areas with high blood lead levels [14, 28]. Current screening rates are now among the highest reported for any established lead screening programs [15].

Before these changes were made, the proportion of eligible children screened each year had declined steadily for a decade [20, 21]. Other long-running lead management and screening programs have reported declines in attendance over time which can be difficult to reverse [29–32]. Nevertheless, dissemination of screening guidelines, highlighting lead risks to local physicians and focussing on children at high risk of lead exposure have increased blood lead screening rates in some communities [33, 34]; the increase achieved in Broken Hill exceeds these.

It is notable that the increased screening participation occurred without additional funding. Many voluntary blood lead screening programs with high participation rates are associated with substantial home remediation programs if children are found to have high blood lead levels [32, 35] or significant outreach programs [36]. The Broken Hill program has not had the funding for either of these since 2001 and has achieved the increased participation within existing resources. While this makes it sustainable from an organisational point of view, there is public frustration about the lack of community-wide action or support to help families whose children have high blood lead levels but who are unable to implement some lead risk reduction activities (e.g., putting down clean soil) because of financial constraints [22]. A recent announcement by the NSW government to invest in a further lead abatement program for Broken Hill has been well received by the community [37].

The effect of the availability of capillary screening varied with age. Attendance of children who were 12 months or younger increased significantly when capillary screening was introduced but there was no change for older children. This differential uptake by different age groups is the reason that overall attendance only increased slightly after capillary screening was introduced.

The largest increase in participation occurred after screening was linked with immunization, which is congruent with previous findings that convenience and cultural appropriateness are critical for optimizing screening rates [34, 38, 39]. However we consider it unlikely that participation would have increased as much as it did if the screening method had not been changed to capillary. Acceptibility to the target population is an important component of screening, and parents are less willing to have children screened if they perceive that the testing method is painful [22, 38]. Moreover, community consultations in Broken Hill had previously identified venous blood collection as contributing to reluctance to have children screened [22]. We conclude that both capillary screening and more convenient access were important for increasing screening participation in Broken Hill.

Increased screening participation does not necessarily indicate increased community awareness of lead as a health issue, or better health outcomes. Nevertheless, while increased screening participation will not in itself reduce community lead risk, it is still an important achievement. Screening provides a point of interaction between health professionals and the community, and importantly the results of screening have confirmed lead is still an important public health issue in Broken Hill and provided the basis for advocacy for increased funding to reduce community lead exposure.

It is unclear how the change to capillary screening affected the estimate of the population blood lead level and proportion of children with blood lead levels ≥5 μg/dL and ≥10 μg/dL. We would have expected a one-off increase in estimates of the mean blood lead level and proportions of children with blood lead ≥5 μg/dL and 10 μg/dL (refs) [40], but this did not happen. Instead these estimates fluctuated at or slightly below the previously observed range. It may be that the increase in estimates that would have been expected from the change in method has been compensated for by accessing a much greater proportion of the population. From a primary prevention perspective, it is important that the capillary method has not given false negatives. The suitability of the current equipment will need to be reviewed in light of the recent reduction of the Australian threshold for investigation of an individual’s lead exposure to 5 μg/dL [41].

The study has two limitations. The first arises from using administrative data sets to calculate denominators. Denominators for the cross-sectional analyses were estimated from the 2005 and 2011 Australian Censuses. We assumed that change between census years was linear but that may not have been the case. Similarly, there is some uncertainlty with the data on the NSW Midwives Data Collection, which was used to estimate denominators for the cohort analysis, due to a time delay in entering data for children born out of state, and the fact it does not account for in/out migrationafter birth. These factors would account for cumulative cohort attendance rates exceeding 100 % in some years.

The use of a before and after study limited our ability to examine for other factors that might have influenced screening rates, and to consider the possibility that offering venous screening at routine visits may have similarly increased attendance. However, attendance rates had been declining steadily since 2001 and the decline stopped the same year capillary screening was introduced. Attendance increased by 5 % over the next 2 years, mainly among infants presenting for their first test (prior to providing screening alongside other well-accepted mainstream programs). Previous evidence [22] suggested the use of venipuncture had resulted in parental reluctance to attend thus we consider the change in screening method was most likely explanation for increased participation in this age group. During the 1990s screening rates peaked during times of increased program activity, but there was no increased program activity during the study period. Similarly because the study was undertaken in one community and there is no control community we can’t rule out broader factors (such as national health promotion campaigns) which could have increased screening, although we are unaware of any such campaigns having occurred during the study period.

## Conclusions

Making changes to a long standing blood lead screening protocol successfully increased participation. Using a screening method acceptable to the community and providing screening alongside other well-accepted mainstream programs for children has the potential to improve participation in other blood lead screening programs. These findings also have implications for improving participation in other health screening programs. Further research is required to assess the effectiveness of this approach in other communities, whether the improved attendance is maintained in the medium to long term, and whether participation rates can be increased to acceptable levels by increasing the convenience of access alone.
